# Report of Committee on Army Legislation1Presented at the Thirty-eighth Annual Meeting of the American Veterinary Medical Association, Hotel Rudolf, Atlantic City, September 5, 1901.

**Published:** 1901-11

**Authors:** D. E. Salmon

**Affiliations:** Chairman, Washington, D. C.


### REPORT OF COMMITTEE ON ARMY LEGISLATION.[1]

By D. E. Salmon, D.V.S., Chairman,
WASHINGTON, D. C.

Your Committee on Army Legislation respectfully report that at the last session of Congress every effort was made to present the subject of suitable recognition for army veterinarians and to secure such legislation as would improve the veterinary service of the United States Army, and at the same time ameliorate the condition of those unfortunate members of our profession who are engaged in that service. The bill, prepared by your committee, creating a veterinary corps and making all veterinarians commissioned officers, was believed to be the best measure that could be advocated. The provisions of this measure were added to the different military bills, and were passed by both houses of Congress, but unfortunately not in the same bill, and were finally eliminated by vote of the Senate. That Congress was at first disposed to grant all that we asked is shown by the favorable action of both houses. It was only after the most active interference of the Secretary of War, and the presentation of letters from certain veterinarians giving the false impression that our measure was not wanted by the army veterinarians or the profession as a whole, that our enemies were able to secure its defeat.

It is unfortunate and humiliating to all respectable members of our profession that on every occasion when there has been an opportunity to secure the recognition of army veterinarians as members of a learned profession there has appeared at the critical moment some renegade whose rank and disgusting treachery has helped to defeat the efforts made for this purpose. It is deplorable that there are men who style themselves veterinarians, but who have neither regard for the honor of the profession nor sympathy for their brethren in the army, who must endure the indignities and insults incident to a position which has neither rank nor

[1] Presented at the Thirty-eighth Annual Meeting of the American Veterinary Medical Association, Hotel Rudolf, Atlantic City, September 5, 1901.

authority. Reproaches are lost on men who are capable of such perfidious action. They should be made to feel the displeasure of the profession in whose front their disloyalty has been so brazenly flaunted. Your committee feel, however, that their labors have not entirely been in vain. The grade of veterinarian of the second class in cavalry regiments has been abolished, and hereafter the two veterinarians in each cavalry regiment will receive the pay and allowance of second lieutenants mounted. This is a great gain. In addition to this it is provided there shall be a veterinarian having the same pay and allowances for each regiment of artillery. On the whole, there will be more veterinarians employed and more money expended for veterinary services than was asked for by your committee.

According to recent press reports, military veterinarians will hereafter wear the uniform of second lieutenants, without the shoulder-straps; their status is assimilated to that of commissioned officers, and they are eligible for detail as members of boards of survey or councils of administration. This will be some mitigation of the conditions which have heretofore prevailed. Your committee have recently seen a photograph of the principal military veterinarians engaged in the campaign in China. Every representative of a foreign army appeared in the uniform of a commissioned officer, with the insignia of his rank, while the poor American veterinarian, sandwiched in between these well-dressed and imposing gentlemen, looks like a teamster or a servant. It is a picture once seen, never forgotten.

The provisions of the new law and the decisions which are alleged to have been made thereunder will, it is hoped, make it impossible for another picture of this kind to be made. It is also hoped the new law may justify a revision of the decision of the Comptroller that military veterinarians are neither officers nor enlisted men, but not a " civil employé."

Finally, your committee, while frankly acknowledging disappointment at the failure of their plan to secure rank and a corps organization, are nevertheless of the opinion that much has been accomplished, that we should feel encouraged, and that the efforts should be continued until complete success is achieved.

